# Alginate nanogels-based thermosensitive hydrogel to improve antidepressant-like effects of albiflorin via intranasal delivery

**DOI:** 10.1080/10717544.2021.1986604

**Published:** 2021-10-07

**Authors:** Dong Xu, Tao Qiao, Yue Wang, Qiang-Song Wang, Yuan-Lu Cui

**Affiliations:** a State Key Laboratory of Component-based Chinese Medicine, Research Center of Traditional Chinese Medicine, Tianjin University of Traditional Chinese Medicine, Tianjin, PR China; b Tianjin Key Laboratory of Biomedical Materials, Institute of Biomedical Engineering, Chinese Academy of Medical Science & Peking Union Medical College, Tianjin, PR China

**Keywords:** Albiflorin nanogels, alginate, thermosensitive hydrogel, intranasal delivery, depression

## Abstract

Depression is a primary public health problem. However, current antidepressants work slowly, and together with side effects. Herein, the alginate nanogels were constructed to load albiflorin (albiflorin nanogels), which further formed albiflorin nanogel loaded self-assembled thermosensitive hydrogel system (albiflorin-NGSTH) and were used to improve its antidepressant effects. The nanogel showed a nano-scaled particle size and stronger antioxidant activity. Rheological studies showed that albiflorin-NGSTH had a sol-gel transition at approximately 28 °C. Albiflorin-NGSTH quickly entered the brain by intranasal delivery, and had a continuously release for albiflorin. Preliminary results of mice behavioral despair tests found that albiflorin-NGSTH had no effects on independent exploratory behavior and anxiety of the mice, and significantly decreased immobility duration of the mice in tail suspension test (TST). Moreover, the intranasally administrated albiflorin-NGSTH at a low dose improved depressive behavior, decreased levels of proinflammatory cytokines, and repaired neuronal damage of chronic unpredictable mild stress (CUMS) rats, which indicated an excellent potential for depression therapy. The treatment of albiflorin-NGSTH on depressive disorder was achieved by regulating signal pathway related to depression. Therefore, albiflorin-NGSTH has an excellent potential for clinical application in intranasal drug delivery systems.

## Introduction

Depression had impacted more than 264 million of all ages in the world. Hence, it is of great clinical value and social significance to strengthen the prevention research of depression and improve the treatment effect (WHO, 2021). Nanocarriers have attracted research attention as drug delivery system since they are an excellent carrier for delivering drug to brain, and helping to attain better therapeutic effects on the central nervous system disease (Craparo et al., 2011). Particularly, there is a good strategy that the nanocarrier targets drug to brain and to enhance the antidepressant effect of drug (Chen et al., 2018). In clinical practice, the most frequently administered antidepressants had side effects and high recurrence rates (Berton & Nestler, 2006; Voican et al., 2014), and acquired about two weeks to work (Mitchell, 2006). As a result, natural polysaccharide-based drug delivery systems provided a promising strategy for drug delivery due to their inherent large surface area, biocompatibility, and low toxicity (Zhang et al., 2019; Vanavil et al., 2020).

It was reported that intranasal delivery had a rapid effect on depressive disorder by nose-to-brain route. At present, intranasal delivery as a noninvasive method in the treatment of neuropsychiatric diseases had received increasing attention, which can deliver drugs to brain and provide a best route for drugs to the brain through the olfactory pathway (Hanson & Frey, 2008; Trotta et al., 2018; Nigam et al., 2019). In 2019, esketamine, the first antidepressant of intranasal delivery, had been approved by the Food and Drug Administration (Cristea & Naudet, 2019), which had the main benefit of the rapid onset for depression. However, the major disadvantage of intranasal delivery was short residence time and drug loss in the nasal cavity because of good fluidity of the solution. At present, some hydrogels with sustained delivery had reported for anti-cancer treatment (Chen et al., 2019; Yang et al., 2020). In particular, the application of thermosensitive and biodegradable hydrogels is a good idea that avoids initial burst release and achieve sustained release of drug (Shi et al., 2021), such as an injectable thermosensitive photothermal-network hydrogel (Liu et al., 2019) and NIR-II light-modulated thermosensitive hydrogel (Ruan et al., 2019). In addition, thermosensitive hydrogel was also used for bioimaging technique or imaging of deep tissues (Chen et al., 2020; Wu et al., 2021). Thermosensitive hydrogel based on PEO-PPO-PEO poloxamers had been reported, which showed a controlled *in situ* release of recombinant adeno-associated viral vectors (Madry et al., 2020). Therefore, combining with the hotspot in current study, alginate nanogels based thermosensitive hydrogel was designed for intranasal delivery of depressive disorder.

Albiflorin as a monoterpene glycoside was the main bioactive component of the root of *Radix Paeoniae* Alba (Ranunculaceae), which might be a traditional Chinese medicine used to treat mental illness (Wang et al., 2016). Albiflorin has a variety of pharmacological activities, which included antioxidant stress (Suh et al., 2013), anti-inflammatory effects (Wang et al., 2014), and neuroprotective effects (Ho et al., 2015). Modern toxicological studies showed that albiflorin had no obvious toxicity and was a safe natural drug ingredient (Han et al., 2018). At the same time, albiflorin also produced antidepressant effects though intervention of multiple targets (Song et al., 2015). Our research results also showed that albiflorin targeted hippocampal phospholipid and tryptophan metabolism for antidepressant-like effects (Wang et al., 2021). However, albiflorin maintained a low blood–brain concentration *in vivo* after oral administration, which was because gut microbiota transformed albiflorin to benzoic acid (Zhao et al., 2018). Furthermore, albiflorin presented a low oral bioavailability (12.09%) (TCMSP, 2012). According to the report that depression with patients had increased levels of markers of inflammation and oxidative stress (Lindqvist et al., 2017), as a result, it was a good choice that albiflorin was used to prepare nanogel-thermosensitive *in situ* hydrogel system that targeted albiflorin to brain and further increased its antidepressant effects.

**Figure 1.**
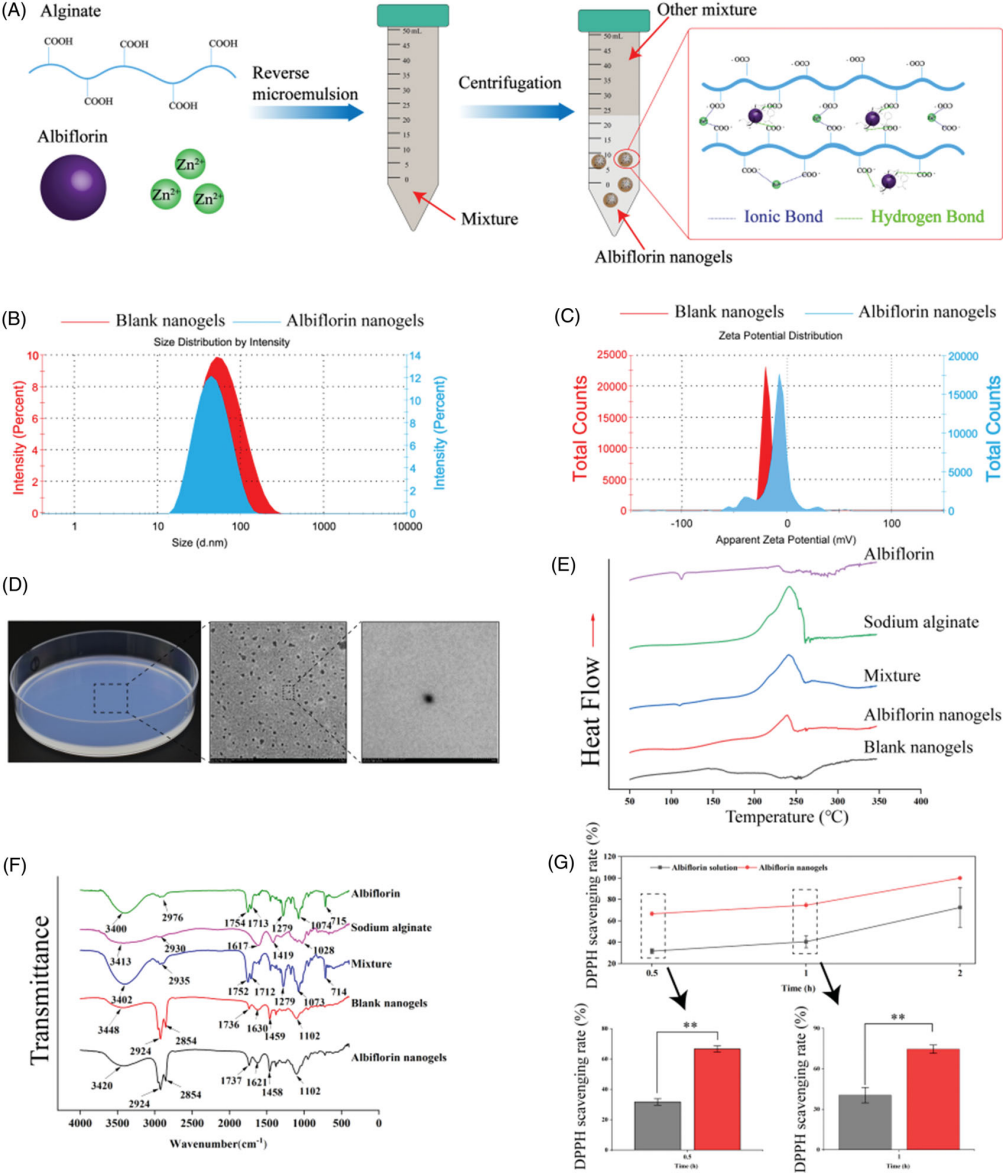
Mechanism and characterization of albiflorin nanogels. (A) Preparation of albiflorin nanogels. (B) Size distribution of albiflorin nanogels. (C) Zeta potential distribution of albiflorin nanogels. (D) Appearance and transmission electron microscope (TEM) microphotographs of albiflorin nanogels. (E) Differential scanning calorimetric thermogram. (F) Fourier transform infrared (FTIR) spectroscopy. (G) Antioxidant activities of albiflorin nanogels. **p< .01, compared with the albiflorin solution.

In this study, we prepared alginate nanogels based thermosensitive hydrogel, which was used as an intranasal drug delivery system. To this aim, the alginate nanogels was constructed to load albiflorin (albiflorin nanogels) via the reverse microemulsion method. Subsequently, the albiflorin nanogels were loaded into thermosensitive hydrogels, which were further investigated *in vitro* and vivo (Figure 1(A)).

## Materials and methods

### Materials

Albiflorin (purity 98%) was obtained from Beijing Ounaer Bioengineering Technology Co., Ltd. (Beijing, China). Sodium alginate (SA, A0682, 100 g) was from Sigma-Aldrich Co. (St. Louis, MO). Span 80, Tween 80, Poloxamer 407 (P 407), and Poloxamer 188 (P 188) were from Sigma-Aldrich Co. (St. Louis, MO). Fluoxetine hydrochloride (Fluo) was bought from Eli Lilly and Company (Suzhou, China). Corticosterone ELISA Kit and Prostaglandin E_2_ ELISA Kit were obtained from Cayman Chemical Company (Ann Arbor, MI). All the other chemicals were of analytical grade.

### Animals

Male SD rats (180–220 g) and male ICR mice (18–20 g) were purchased from the Vital River Laboratory Animal Technology Co., Ltd. (Beijing, China). Male SD rats and male ICR mice were fed at 24 ± 1 °C and relative humidity (56 ± 5%). Procedures of the animal experiment were consistent with the Guidelines for Care and Use of Experimental Animals. Experimental protocols were permitted by the Animal Ethics Committee of Tianjin University of Traditional Chinese Medicine (approval number: TCM-2018-038-E12; TCM-LAEC2021124). All experiments were reported under the Animal Research: Reporting In Vivo Experiments (ARRIVE) guidelines, which also was consistent with the recommendations for good publishing practice in physiology (du Sert et al., 2020).

### Preparation of albiflorin nanogels

Albiflorin nanogels were synthesized with the reverse microemulsion method (Qi et al., 2015). In brief, Span 80 (1.05 mL) and Tween 80 (0.45 mL) were combined into liquid paraffin (150 mL) with stirring of 500 rpm (EURO-ST IKA, Staufen, Germany) for 30 min. Sodium alginate solution (0.5%, w/v) was obtained by dissolving the SA in deionized water with magnetism stirring for 6 h at 45 °C. In the following process, an amount of 45 mL SA solution was added dropwise into the above oil phase with stirring of 1000 rpm for 60 min. Next, 15 mL ZnCl_2_ solution containing 0.5% of albiflorin (w/v) was added, and continuously stirred for 1 h to form uniform-sized droplets. Finally, the lower layer was obtained using centrifugation. Nanogels without albiflorin as control were also prepared by the above methods.

### Characterization of albiflorin nanogels

Size and zeta potential of albiflorin nanogels were determined by particle size analyzer (NanoZS, Malvern Instruments, Malvern, UK), which was based on a method of phase-analysis light scattering (PALS). Morphology of nanogels was observed using transmission electron microscope (TEM) (H-7650, Hitachi, Tokyo, Japan). Differential scanning calorimetry (DSC) curves of albiflorin nanogels were obtained by DSC (Jade DSC, Perkin-Elmer Corp, Wilton, CT). FT-IR spectra of albiflorin nanogels were obtained by Thermo Nicolet iS10 FT-IR spectroscopy (Thermo Scientific, Waltham, MA).

### Drug loading rate of albiflorin nanogels

Briefly, albiflorin was extracted form albiflorin nanogels by ultrasonic extraction and centrifugation. The supernatant was filtered using a filter with a pore size of 0.45 μm. The amount of albiflorin was determined using a 2695 HPLC system (Waters, NY) with a Kromasil 100-5 C_18_ column (250 × 4.6 mm, 5 μm, Sweden) at room temperature (25 °C). The mobile phase included water (65%) and methanol (35%), and the detection was conducted at 230 nm. Drug loading efficiency of albiflorin nanogels was calculated using the following formula:

$$\text{Drug loading rate }(\%)=\;\frac{\text{amount of albiflorin in nanogels}}{\text{amount of nanogels}}\;\times 100\%$$

### Antioxidant activities of albiflorin nanogels

The DPPH radical scavenging assay was performed using the previous methods that had been reported (Rufino et al., 2009). The test samples (including albiflorin solution and albiflorin nanogels, 3.6 mg/mL calculated by the weight of albiflorin in methanol or nanogels) and DPPH were dissolved in methanol as *A_i_*. The methanol was used to replace DPPH as *A*_0_. The mixture was added into the 96-well plate, and three parallel operations were done. The mixture was reacting in dark at 25 °C for 15 min. The methanol and DPPH were mixed as A_max_. OD values of all samples were measured at 517 nm. The DPPH radical scavenging rate was calculated as follows:

$$\text{DPPH scavenging rate\%}=(1-\;\frac{A_{i}-A_{0}}{A_{\max}})\times 100\%$$

### Preparation and characteristic of albiflorin-NGSTH

First, precooled normal saline and albiflorin nanogels with the settled formulation ratio were mixed with stirring of 800 rpm for 4–6 h at 4 °C. Second, P 407 and P 188 were added into vial to form albiflorin-NGSTH at 4 °C. In the end, albiflorin-NGSTH as a stock solution was used to the next study. The optimization of different ratio of P 407 and P 188 is reported in Table 1.

**Table 1.** Optimization of different ratio of P 407 and P 188.

Batches	Albiflorin nanogels (w/w, %)	P 407 (w/w, %)	P 188 (w/w, %)	Normal saline (w/w, %)
1	12	14	0	74
2	12	14	1	73
3	12	14	2	72
4	12	16	0	72
5	12	16	1	71
6	12	16	2	70
7	12	18	0	70
8	12	18	1	69
9	12	18	2	68

The optional formulation of albiflorin-NGSTH was screened by the tube inversion method. The intelligent thermostat metal bath was used to control temperature of the tubes containing stock solutions. Temperature of the stock solutions were altered at an increase of 1 °C/min. Gelling temperature of the stock solutions was detected using tube inversion method. The temperature that the solutions kept immobile was recorded, as the gelation temperature. Each batch was analyzed in triplicate.

The rheological analysis of the stock solutions was performed by the rheometer (MCR 302, Anton-Paar, Graz, Austria). Range of the temperature was set at 20–40 °C. Frequency and shear stress were adjusted to 1 Hz and 0.01 Pa, respectively. The stock solutions of 0.5 mL were detected by rheometer. Finally, rheological properties of the stock solutions were analyzed.

### Release behavior of albiflorin-NGSTH

*In vitro* release behavior of albiflorin-NGSTH was carried out in phosphate-buffered saline (PBS, pH 6.4). Briefly, the gel stock solution (2 mL) was added into a dialysis bag, and then suspended in PBS. The release behavior of albiflorin-NGSTH was performed on an incubator shaker with stirring of 55 rpm at 37 °C (Darwin, 2019). At the specified time, the release samples of 2 mL were collected from the release medium. Meanwhile, 2 mL of fresh PBS was added into release medium. The release samples were detected using HPLC with a wavelength of 230 nm. Each batch was analyzed in triplicate.

### *In vivo* tissue distribution of albiflorin-NGSTH

According to previous research methods (Deng et al., 2019), rhodamine B was used to label the nanogels. Herein, rhodamine B-labeled albiflorin-NGSTH was prepared by the rhodamine B-labeled albiflorin nanogels and poloxamer. *In vivo* distribution of the rats was detected by fluorescence imaging. Male SD rats (220–240 g) were given to rhodamine B-labeled albiflorin-NGSTH by intranasal delivery. Next, *in vivo* distribution (including the brain, liver, spleen, and kidney) was observed by the Maestro system (CRI, Woburn, MA) at 30, 60, 120, 240, 480, 720, and 1440 min.

### Antidepressant activities of albiflorin-NGSTH in mice behavioral despair tests

A total of 96 mice were randomly divided into eight groups. There are control group (normal saline, intranasal delivery), fluoxetine group (positive drug, 10 mg/kg, gavage), albiflorin solution group (7 mg/kg, gavage, i.g.), albiflorin solution group (1.75 mg/kg, intravenous (i.v.) injection), albiflorin nanogel (0.35 mg/kg calculated by the weight of albiflorin in albiflorin nanogels, intranasal delivery), and albiflorin-NGSTH (0.175, 0.35, and 0.7 mg/kg calculated by the weight of albiflorin in albiflorin nanogels, intranasal delivery). According to our previous methods, the exploratory behavior and locomotor activity of the mice were investigated by behavioral equipment (Multifunctional 256,060 Series, TSE Systems, Bad Homburg vor der Höhe, Germany), which included number of rearings, total distance, and activity time of the mice. Tail suspension test (TST) of the mice was further recorded and analyzed by the above behavioral equipment (Xu et al., 2020, 2021).

### Establishment of chronic unpredictable mild stress (CUMS) model

CUMS rats model was based on a previous method with slight modification (Willner et al., 1987). Experimental rats were randomly divided into control group (normal saline, intranasal delivery), CUMS group (normal saline, intranasal delivery), fluoxetine group (positive drug, 10 mg/kg, i.g.), albiflorin solution group (7 mg/kg, i.g.), albiflorin solution group (1.75 mg/kg, i.v.), and albiflorin-NGSTH (0.175, 0.35, and 0.7 mg/kg calculated by the weight of albiflorin in albiflorin nanogels, intranasal delivery). All rats underwent CUMS protocol for 5 weeks, except for the control group. Experimental rats were administrated in the last seven days of the CUMS protocol. The CUMS protocol included multiple stimuli, namely, swimming in ice water, food, and water deprivation within 24 h, 10 min of light stimulation, electrical stimulation (36 mV, 10 sec each time), 2 min of oscillation and tail clip. The weight change and sugar water preference of the rats were recorded during the CUMS protocol.

### Open field test (OFT) of CUMS model

After the CUMS protocol and the administration were performed, locomotor activity of the rats was evaluated using the OFT. Behavioral characteristics (including total distances, number of rearings, activity time) were observed and recorded using animal behavioral video analysis system (Multifunctional 256060 Series, TSE Systems, Bad Homburg vor der Höhe, Germany).

### Biochemical indicators and pathological changes of rats

All rats were sacrificed after the OFT. Plasma of the rats were obtained by centrifuging at 3000×*g* for 10 min. Corticosterone and prostaglandin E_2_ (PGE_2_) of the rats in the plasma were detected using enzyme-linked immunosorbent assay (ELISA). The hippocampi of the rats were obtained from the brain, and 10% neutral formalin was used to fix the hippocampi. Hematoxylin and eosin (H&E) were used to stain tissue after the fixed tissue undergo a series of treatments that included dehydration, embeddedness, and sectioning. The light microscope (OLYMPUS, Tokyo, Japan) was used to observe the stained sections.

### RNA-seq of hippocampus transcriptome

Total RNA was obtained from the hippocampi of rats using Trizol reagent kit according to the manufacturer’s instructions. RNA degradation and contamination were monitored on 1% agarose gels and its purity was checked using the Nano Photometer spectrophotometer (IMPLEN, Westlake Village, CA). RNA concentration was measured by Qubit RNA Assay Kit in Qubit 2.0 Flurometer (Life Technologies, Carlsbad, CA), and its integrity was determined by the RNA Nano 6000 Assay Kit of the Bioanalyzer 2100 system (Agilent Technologies, Santa Clara, CA). Sequencing libraries were generated using NEBNext Ultra RNA Library Prep Kit for Illumina (NEB, Ipswich, MA) following manufacturer’s recommendations and index codes were added to attribute sequences to each sample. At last, PCR products were purified (AMPure XP system) and library quality was assessed on the Agilent Bioanalyzer 2100 system. The cDNA libraries were sequenced on an Illumina HiSeq platform according to standard sequencing protocol. Differential expression analysis of albiflorin-NGSTH and CUMS group was performed using the DESeq2 R package (1.16.1). The resulting *p* values were adjusted using the Benjamini and Hochberg’s approach for controlling the false discovery rate. Genes with an adjusted *p* value <.05 found by DESeq2 were assigned as differentially expressed. Gene Ontology (GO) enrichment analysis of differentially expressed genes was implemented by the cluster Profiler R package, in which gene length bias was corrected. GO terms with corrected *p* value less than .05 were considered significantly enriched by differential expressed genes. The cluster Profiler R package was used to test the statistical enrichment of differential expression genes in KEGG pathways.

### Statistical analysis

All data were analyzed using Origin Pro 2021 (OriginLab, Northampton, MA). Data were expressed as the means ± standard error (mean ± SE). One-way analysis of variance (ANOVA) was used to analyze between multiple groups. A *p*< .05 or *p*< .01 was considered as statistically significant difference.

## Results and discussion

### Preparation and characterization of albiflorin nanogels

Preparation of albiflorin nanogels is shown in Figure 1(A). Results of size and zeta potential are demonstrated in Figure 1(B,C), respectively. The size distribution of nanogels was randomly measured, and the obtained data represented that albiflorin nanogels and blank nanogels were 45.6 ± 5.2 nm and 49.4 ± 0.0 nm, respectively. Compared to blank nanogels, the particle size of albiflorin nanogels was reduced, which might be due to a greater intermolecular force that albiflorin participated in the mechanism of the nanogels by hydrogen bonding. Polydispersity index (PDI) that described the particle size distribution of the nanogels was less than 0.20, which indicated that albiflorin nanogels had a narrow particle size distribution (Willner et al., 1987). In terms of zeta potential, surface charge density shows the electric potential (Sikora et al., 2016). Both blank nanogels and albiflorin nanogels were negatively charged with zeta potential of about −10.0 ± 0.3 mV and −19.8 ± 0.9 mV, respectively. Therefore, the absolute value of zeta potential was increased by addition of albiflorin, which suggested that albiflorin might improve the stability of nanogels by a stronger intermolecular force. At the same time, albiflorin nanogels displayed a pale blue color under the LED lights. TEM microphotographs presented that the nanogel was almost subspherical shape (Figure 1(D)). The experimental results demonstrated that content of albiflorin in alginate nanogels was 2.43% by HPLC analysis, which was suitable for intranasal delivery of depression therapy.

The physical status of SA, albiflorin, SA-albiflorin-ZnCl_2_, blank nanogels, and albiflorin nanogels was examined by DSC (Figure 1(E)) and FTIR (Figure 1(F)). Albiflorin nanogels did not show a characteristic peak of albiflorin on the FTIR compared to free albiflorin. Results of the DSC showed that albiflorin nanogels, compared to albiflorin, did not appear an endothermic peak or exothermic peak of albiflorin. The above curves indicated that albiflorin might participate in the structure of alginate nanogels, which might include physical inclusion and hydrogen bonding. Nevertheless, because of the low drug loading rate of alginate nanogels and the weak force between the compounds, some characteristic peaks such as the hydrogen bond of alginate and albiflorin were not be found on the infrared spectrum.

Antioxidant activities of albiflorin nanogels are shown in Figure 1(G). It was observed that, compared to albiflorin solution, albiflorin nanogels had a higher DPPH scavenging rate, and presented strongly antioxidant activities on free radicals. It was reported that Zn^2+^ had been selected as a cross-linker cation because of its antioxidant properties, and its supplementation was considered as an antioxidant to protect cells (DiSilvestro, 2000; Szuster-Ciesielska et al., 2009; Varghese et al., 2009). Sodium alginate, isolated from brown algae, also exhibited important antioxidant activity (Sellimi et al., 2015). Therefore, the increase of antioxidant activities of albiflorin nanogels may be related to Zn^2+^, SA, and zinc alginate.

### Preparation and characterization of albiflorin-NGSTH

**Figure 2.**
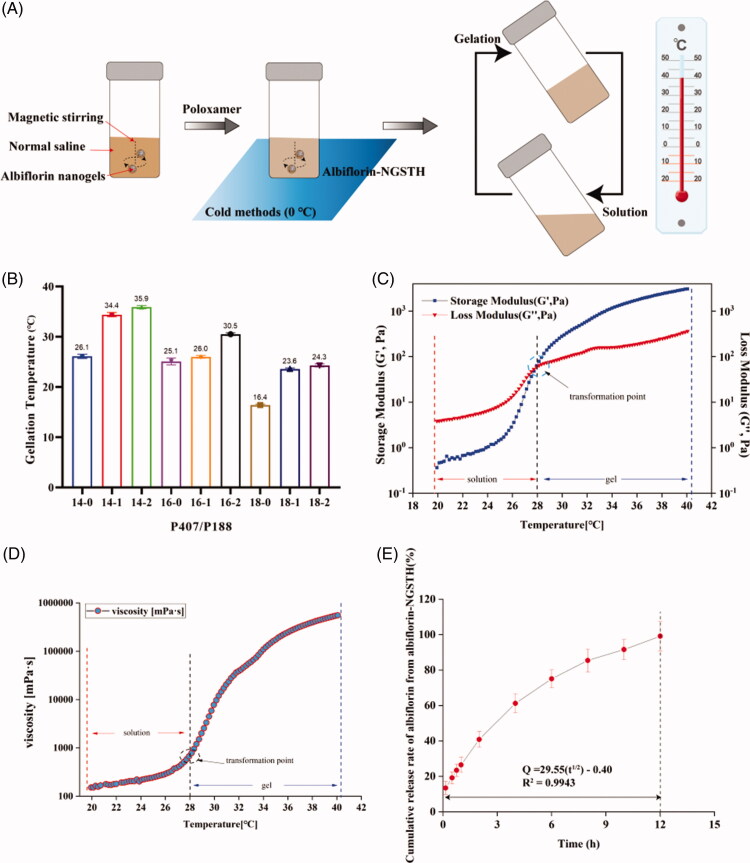
Preparation and characterization of albiflorin-NGSTH. (A) The preparation process of albiflorin-NGSTH. (B) Gelling temperature of the formulations. (C) Rheological analysis of the optimal prescription (P 407/P 188, 16/2). (D) Viscosity of the optimal prescription. (E) *In vivo* release of albiflorin-NGSTH.

The preparation process of albiflorin-NGSTH is shown in Figure 2(A). After the addition of poloxamer, albiflorin-NGSTH presented a temperature-sensitive property under the condition of low temperature and high temperature. This was because polyoxypropylene oxide, as hydrophobic units of poloxamer, produced dehydration and aggregation, so formed a dense and ordered grid. Poloxamer tightly encapsulated the albiflorin-NGSTH in the interior. Meanwhile, the polyoxyethylene oxide shell also rapidly generated gelation *in vivo.* Therefore, poloxamer sustained release of albiflorin and increased the local drug concentration by self-assembly characteristic.

Initially, the optimal formulation that was adapt to intranasal delivery was screened by the tube inversion method. It was observed that with the addition of P 407, the gelation temperature decreased gradually, and P 188 was the opposite in Figure 2(B). When the ratio of P 407 and P 188 was close to 16/2, the gelling temperature (30.5 °C) conforms to intranasal administration. Gelling temperature of the optional formulation was further confirmed by rheometer (Figure 2(C)). The point of intersection of storage modulus (*G*′) and loss modulus (*G*″) curve was as the final gelation temperature. It was found that the dramatic increase of the *G*′ and *G*″ at approximately 28 °C indicated gelling temperature of albiflorin-NGSTH. Furthermore, the viscosity of the albiflorin-NGSTH also was increased significantly by the change of temperature, which indicated that albiflorin-NGSTH had mucoadhesive properties (Figure 2(D)).

*In vitro* drug release of albiflorin in albiflorin-NGSTH is shown in Figure 2(E). The release results showed that the accumulative release rate of albiflorin from albiflorin-NGSTH could reach near 100% in the first 12 hours in the PBS (pH 6.4). The release behavior of albiflorin-NGSTH could last for 12 hours, implying that albiflorin was well encapsulated into the nanogels. Furthermore, albiflorin-NGSTH may prolong the retention time of albiflorin in the brain, and increase the potential neuroprotection time for the focal area of brain. Meanwhile, different release mechanisms were proposed for albiflorin-NGSTH releasing in PBS. Based on the values of *R*^2^ in Table 2, the release process of albiflorin-NGSTH most likely fits to the Higuchi and Ritger–Peppas equation, which suggested that the release mechanism of albiflorin-NGSTH not only presented a sustained and controlled property for albiflorin, but might be due to drug diffusion governing the drug release.

**Table 2.** Models and equations of albiflorin releasing from albiflorin-NGSTH in PBS.

Model	Equation	*R* ^2^
Zero-order	*Q* = 7.36*t* + 20.93	0.9414
First-order	*Q* = 98.01 (1 – e^–0.27^*^t^*)	0.9731
Higuchi	*Q* = 29.55*t*^1/2^ – 0.40	0.9943
Ritger–Peppas	*Q* = 29.32*t*^0.5^	0.9943

### Fluorescence distribution of albiflorin-NGSTH

**Figure 3.**
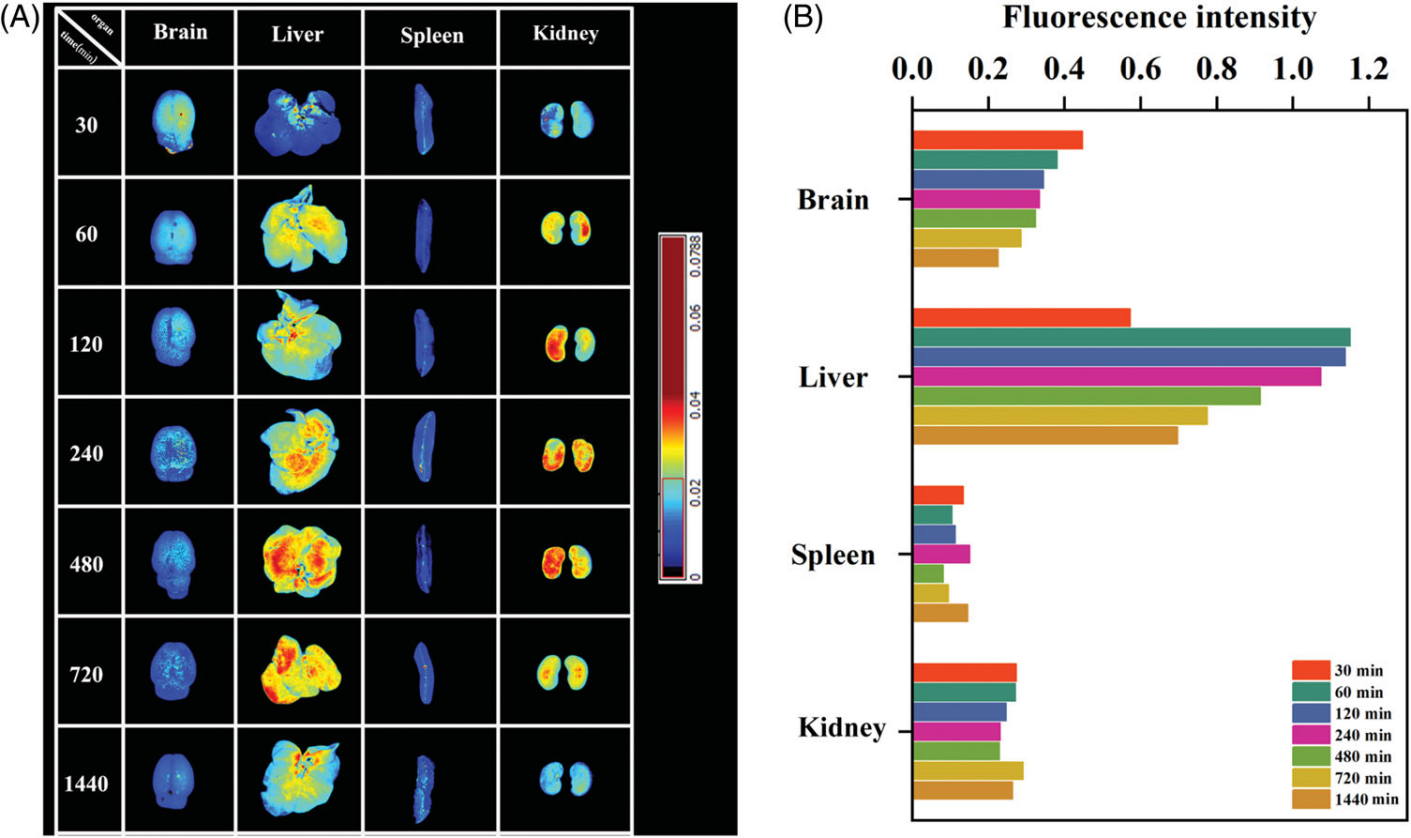
*In vivo* tissue distribution of albiflorin-NGSTH. (A) Fluorescence distribution of albiflorin-NGSTH. (B) Fluorescence intensity analysis of organs.

*In vivo* distribution and fluorescence intensity analysis of rhodamine B-labeled albiflorin-NGSTH in rats are shown in Figure 3(A,B), respectively. It was observed that rhodamine B-labeled albiflorin-NGSTH was intranasally administered after 30 min; the fluorescence intensity of the brain was the most obvious compared to later time. The above results showed that nanogels can fast penetrate the brain by nose-to-brain route and predominantly accumulate in the brain. Liver and kidney also showed stronger fluorescence, which indicated that most of the drugs were absorbed into the blood through the nasal mucosa and further distributed to the liver and kidneys. By 1440 min, brain of the rat presented a weak fluorescence, which indicated that albiflorin-NGSTH exhibited a long and sustained release for albiflorin. At the same time, fluorescence gradually disappeared at the other organs. Due to smaller size of nanogels, large quantities of albiflorin nanogels accumulated in the liver and were eventually excreted by the kidneys. Therefore, these results confirmed the rapidly antidepressant effects of albiflorin-NGSTH, and it can continuously release albiflorin.

### Antidepressant activities of albiflorin-NGSTH in mice behavioral despair tests

**Figure 4.**
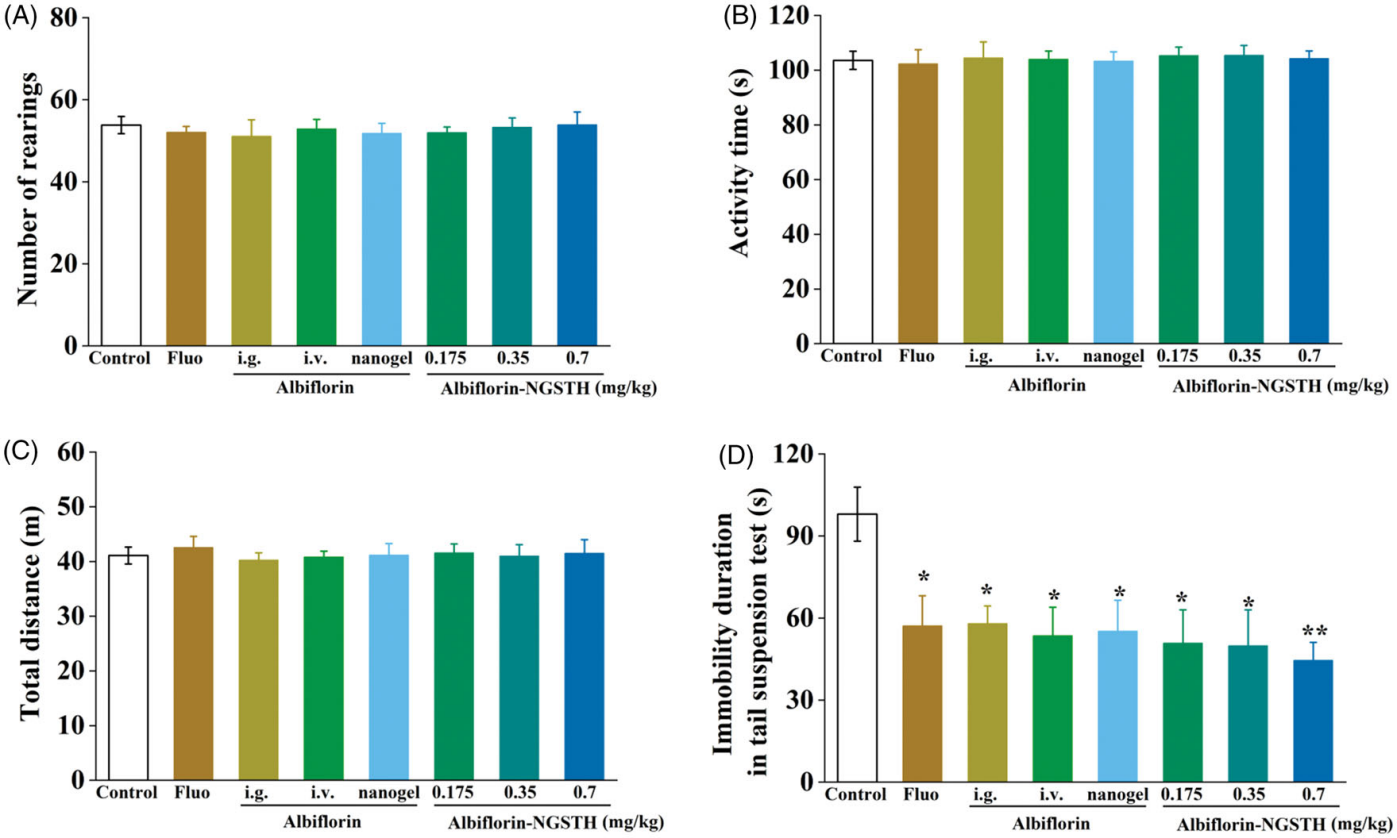
Antidepressant activities of albiflorin-NGSTH in mice behavioral despair tests. (A) Number of rearings of the mice. (B) Activity time of the mice. (C) Total distance of the mice. (D) Immobility duration of the mice. *p< .05 or **p< .01, compared with the control group.

In order to evaluate whether albiflorin-NGSTH effects on independent exploratory behavior and anxiety of the mice, open filed test (OFT) was performed in open field apparatus. It is observed in Figure 4(A–C) that each administration group, compared to control group, had no significant difference. These results showed that albiflorin and its preparation had no effects on independent exploratory behavior and anxiety of the mice.

As shown in Figure 4(D), antidepressant activities of albiflorin-NGSTH were further evaluated by TST, a method that screened and assessed new potential antidepressant drugs (Willner, 1984). It was found that the intranasally administrated albiflorin-NGSTH was lower than that of fluoxetine solution and albiflorin solution administrated by intragastric or i.v. administration, but albiflorin-NGSTH significantly decreased immobility duration of the mice in TST and reversed behavioral despair of the mice. Compared to albiflorin nanogel, albiflorin-NGSTH showed the lower immobility duration, which indicated that self-assembled thermosensitive hydrogel system helped to increase antidepressant activities of albiflorin nanogel. These experimental results demonstrated superiority of albiflorin-NGSTH administrated by nasal cavity compared to oral administration and necessity of self-assembled thermosensitive hydrogel system on the nanogel that was used to intranasal delivery. Therefore, antidepressant effects of albiflorin-NGSTH will be mainly studied in the further experiment.

### Pharmacodynamic study of albiflorin-NGSTH on CUMS rats

**Figure 5.**
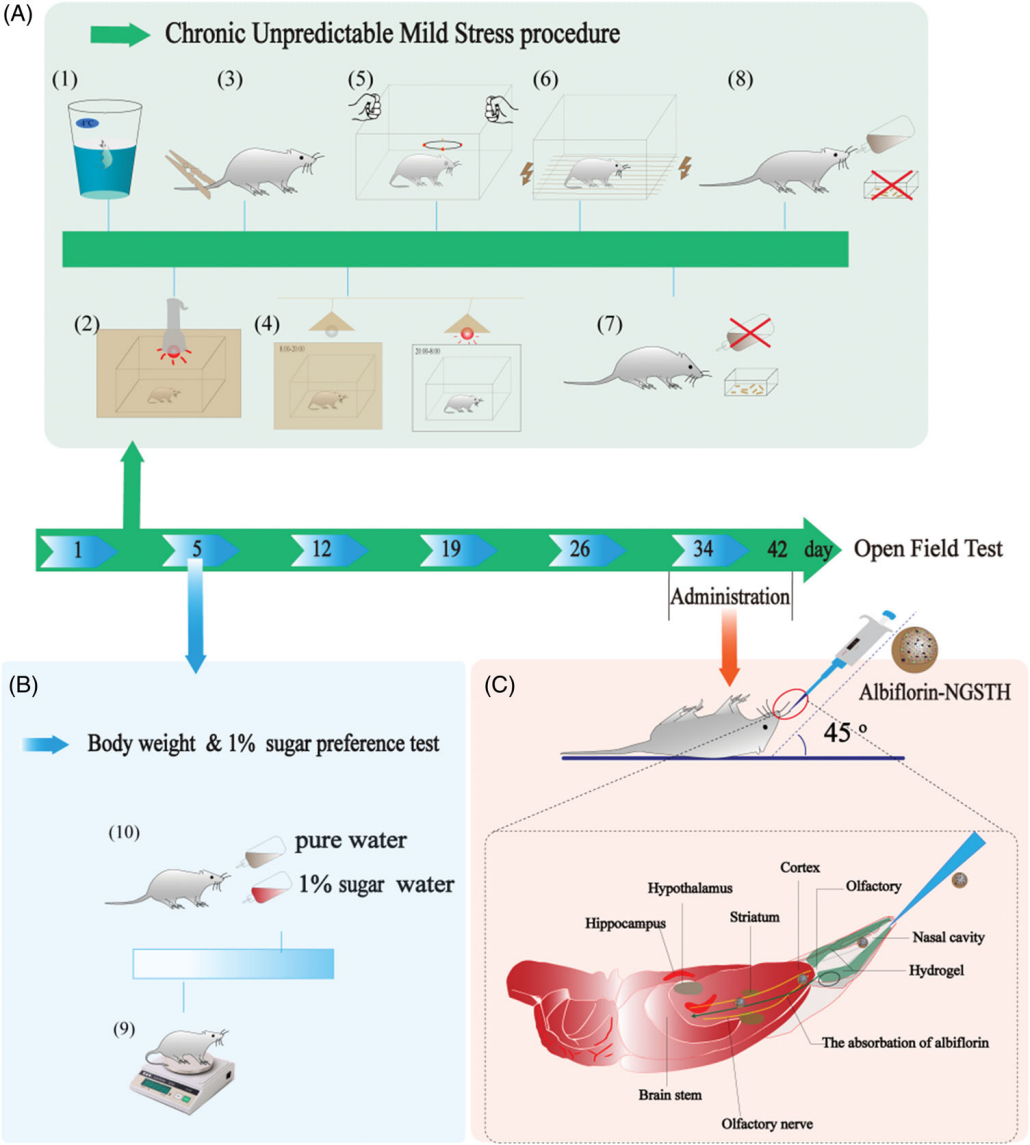
The procedure of CUMS model and intranasal delivery. (A) The procedure of CUMS model. (1) Swimming in ice water. (2) Light stimulation. (3) Tail clip test. (4) Reverse of day–night cycle. (5) Oscillation. (6) Electrical stimulation. (7) Water and (8) food deprivation. (B) The observation of (9) body weight and (10) sugar preference test. (C) Intranasal delivery of albiflorin-NGSTH.

**Figure 6.**
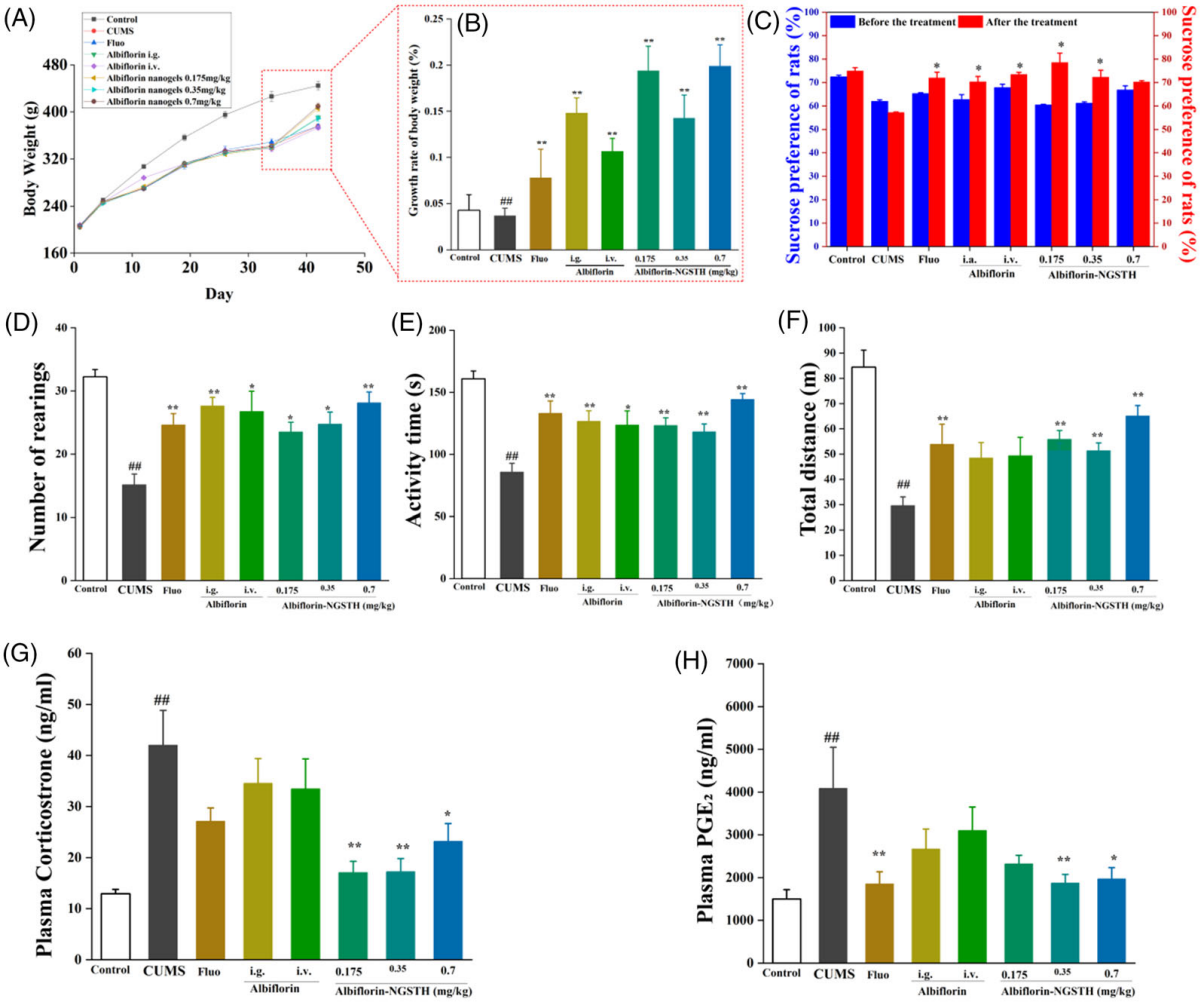
Pharmacodynamic study of albiflorin-NGSTH on the CUMS rats. (A) Change of body weight of the rats. (B) Growth rate of body weight of rats. (C) Sucrose preference of rats. (D) Number of rearings of the rats. (E) Activity time of rats. (F) The total distance of rats. (F) Corticosterone concentration in plasma of rats. (H) PGE_2_ concentration in plasma of rats. ^##^*p* <.01, compared with the control group. *p <.05 or **p <.01, compared with the CUMS group.

CUMS model was widely used as the study of depressive behavior (Gronli et al., 2005; Nirmal et al., 2008). CUMS model is shown in Figure 5. In the experiment, CUMS significantly decreased the body weight of rats (Figure 6(A)), growth rate of body weight (Figure 6(B)), and sucrose preference of rats (Figure 6(C)). However, albiflorin-NGSTH reversed the above adverse effects. In the behavior test of rats, albiflorin-NGSTH increased low number of rearing (Figure 6(D)), low activity time (Figure 6(E)), and short total distance (Figure 6(F)) induced by CUMS model. However, albiflorin-NGSTH was less than the dose of the other group. These results further indicated the advantage of albiflorin-NGSTH.

Meanwhile, some studies had demonstrated that depressive patients had an abnormal of inflammation and HPA axis (Druzhkova et al., 2019), and these abnormal changes were improved by antidepressant drugs (Wang et al., 2011). In this study, PGE_2_ and corticosterone as indicators of proinflammatory cytokines and HPA axis respectively were reversed using albiflorin-NGSTH. Albiflorin-NGSTH decreased corticosterone levels (Figure 6(G)) and PGE_2_ levels (Figure 6(H)). Albiflorin-NGSTH, compared to the oral albiflorin solution, better reversed corticosterone and PGE_2_ levels. Compared to i.v. albiflorin, intranasally administrated albiflorin-NGSTH suggested an advantage of delivery route. To sum up, albiflorin-NGSTH can increase body weight and sucrose preference of rats, improve abnormal behavior of CUMS model, and reserve high plasma levels of corticosterone and PGE_2_ of CUMS model. Experimental results showed that albiflorin-NGSTH, a low dose, had good antidepressant effects on the CUMS model.

### Repairing effect of albiflorin-NGSTH on hippocampus injury of CUMS model

**Figure 7.**
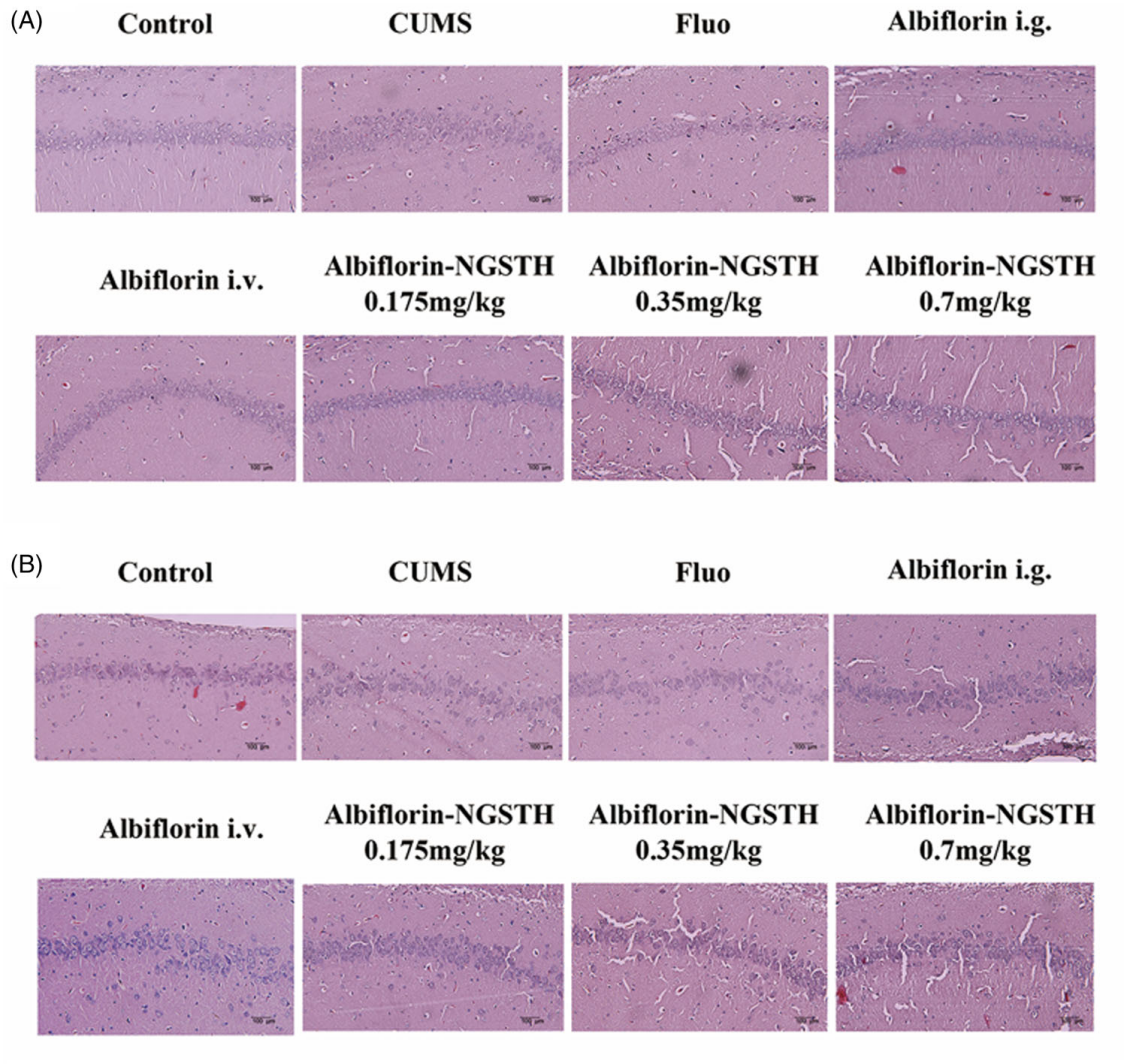
Pathological changes of rat hippocampus. (A) Rat hippocampal CA1 subregion. (B) Rat hippocampal CA3 subregion. Scale bar: 100 µm.

The hippocampus is considered an important region for the development of depression, which was also related to the growth of learning and memory (Fasick et al., 2015). It was observed that neuronal atrophy of the hippocampus showed the dysfunction of cellular processes, which were consistent with characteristics of clinically depressed populations (Morales-Medina et al., 2017). Morphological changes of the neurons were detected using H&E staining to evaluate the antidepressant effect of preparations. Results of staining in the hippocampus are indicated in Figure 7(A,B). It was observed that there was a significant change of pyramidal cells. Compared to the CUMS model, pyramidal cells of albiflorin-NGSTH in CA1 and CA3 subregion showed a regular and tidier cell arrangement, which was close to normal cells. Although intragastric administration of fluoxetine and albiflorin, and tail i.v. injection of albiflorin presented a similar effect in the treatment of CUMS model, but the dose of albiflorin-NGSTH was very small. Therefore, albiflorin-NGSTH improved the morphology of neuronal cells, which presented a better antidepressant effect at a low dose in the CUMS model.

### Transcriptome studies of albiflorin-NGSTH on hippocampus of CUMS model

**Figure 8.**
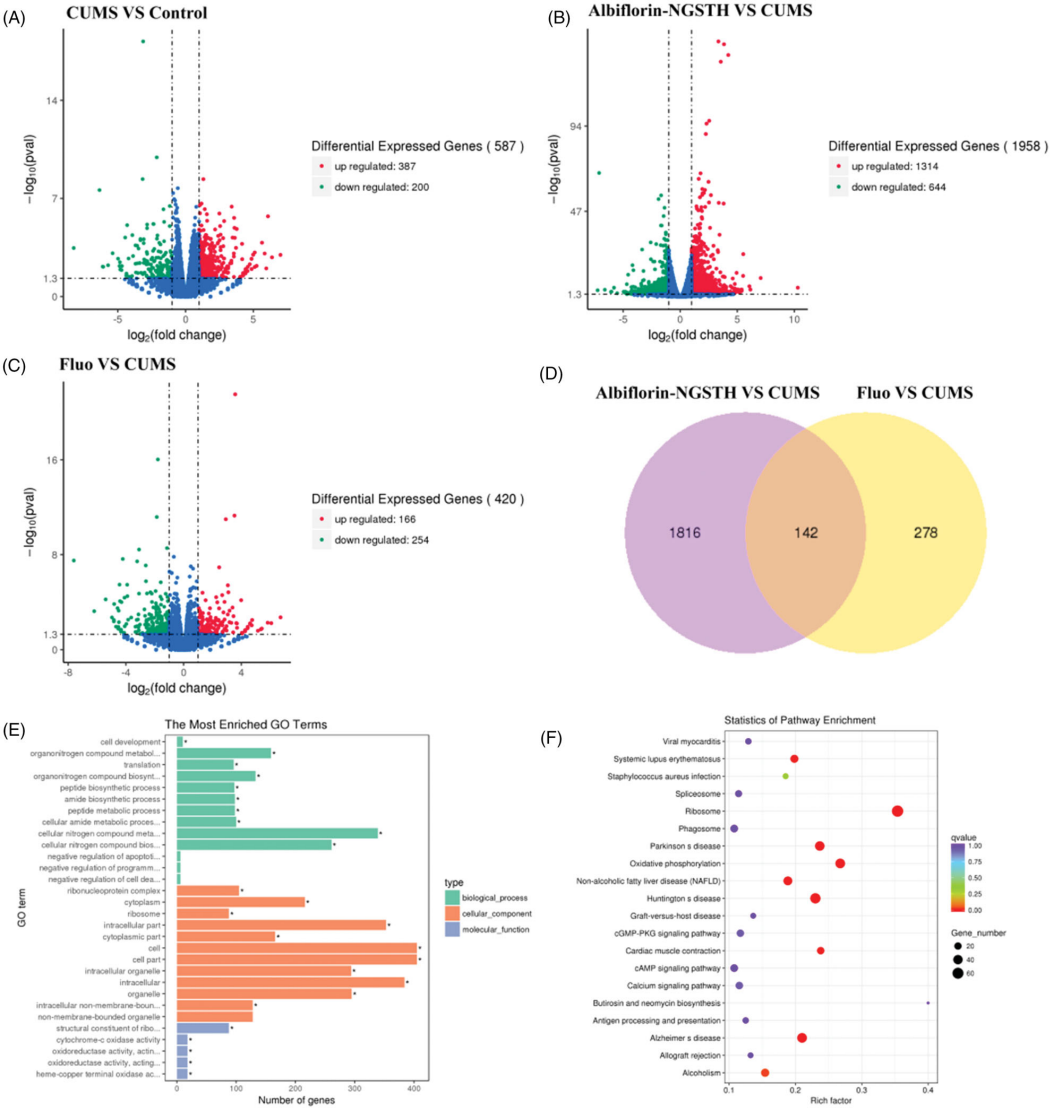
Transcriptome analysis in the hippocampus of rats. Volcano map: (A) CUMS model compared with control group, (B) albiflorin-NGSTH compared with CUMS model, and (C) fluoxetine group compared with CUMS model. (D) Venn diagram of comparison of albiflorin-NGSTH and fluoxetine. (E) GO enrichment analysis of antidepressant effects of albiflorin-NGSTH. (F) KEGG pathway enrichment analysis of antidepressant effects of albiflorin-NGSTH.

Differentially expressed genes were shown in the volcano map, which included the comparison between CUMS model and control group (Figure 8(A)), the comparison between albiflorin-NGSTH and CUMS model (Figure 8(B)), and the comparison between fluoxetine and CUMS model (Figure 8(C)). It was observed that CUMS significantly regulated the differential expressed genes of control group, and caused a depressive behavior of rats. Differentially expressed genes were prominently regulated by administration of albiflorin-NGSTH and fluoxetine. Albiflorin-NGSTH had a widely regulation for differential expressed genes according to the results of volcano map and Venn diagram (Figure 8(D)).

Gene Ontology is an international standard classification system for gene function, and differentially expressed genes were classified according to cellular component, molecular function, and biological process. KEGG is also the classic public database of pathways (Fang et al., 2020). The above methods were performed using gene function analysis and pathway analysis in the present study. The distribution of target genes in enriched GO functions in the albiflorin-NGSTH vs. CUMS model is shown in Figure 8(E). KEGG enrichment analysis was performed to screen for a significantly enriched KEGG pathway (Figure 8(F)). From the point of view of biological pathway, the specific pathway was found with significant difference in expression between groups through the enrichment analysis of differential expressed genes. Enrichment analysis of GO and KEGG showed that differentially expressed genes induced by CUMS were involved in the regulation of multiple neural pathways, including cAMP signal pathway, calcium ion signal pathway and cGMP PKG signal pathway. It was reported that cAMP signal pathway, calcium ion signal pathway and cGMP PKG signal pathway were related to the incidence of depression (Armstrong et al., 2009; Bergantin, 2020). Therefore, the results of pharmacodynamics and transcriptomics showed albiflorin-NGSTH had a good antidepressant effect on depressive disorder through the regulation of cAMP signal pathway, calcium ion signal pathway, and cGMP PKG signal pathway.

## Conclusions

In this study, alginate nanogel was prepared using reverse microemulsion method. Next, alginate nanogel loaded with albiflorin was wrapped into thermosensitive hydrogel for intranasal delivery of albiflorin. The results showed that the prepared albiflorin nanogel met the characteristics of nano-preparation and presented stronger antioxidant activities. The gelling temperature of albiflorin-NGSTH conformed to intranasal delivery. Experimental results showed that albiflorin-NGSTH can adhere to the nasal mucosa for continuous release of albiflorin and rapidly enter to the brain in the first 30 min. Results of mice behavioral despair tests showed that albiflorin-NGSTH had no effects on independent exploratory behavior and anxiety of the mice, and significantly decreased immobility duration of the mice in TST. Compared to other administration group, albiflorin-NGSTH at a low dose effectively improved the abnormal behavior and biochemical indicators of CUMS model, and showed a good antidepressant efficacy and preparation superiority. Antidepressant effects of albiflorin-NGSTH were mainly achieved by regulating oxidative phosphorylation, cAMP, calcium ion, and cGMP PKG pathway.
